# Thumb Reconstruction Using a Modified Masquelet Technique Following Crush Injury: A Case Report

**DOI:** 10.5704/MOJ.2603.019

**Published:** 2026-03

**Authors:** MK Ahmad-Faris, G Vijayan, R Ankimtay

**Affiliations:** 1 Department of Orthopaedic Surgery, Miri Hospital, Miri, Malaysia; 2 Department of Orthopaedics and Traumatology, Sarawak General Hospital, Kuching, Malaysia

**Keywords:** thumb reconstruction, masquelet technique for thumb, thumb crush injury, thumb salvage surgery, two-stage technique for thumb

## Abstract

Traumatic crush injury of the thumb is devastating and often resulted in poor functional outcome. Various reconstruction options available according to surgical expertise. Masquelet technique is well-established in the long bones of lower limb. Only a handful of cases reported for thumb, especially in Asia region. We described a case of crush injury of right thumb following a trauma. The bony defect was initially filled with antibiotic spacer and subsequent reconstruction with tricortical iliac graft, a modified Masquelet two-stage technique. It is a versatile tool which provide satisfactory functional outcome and hand appearance.

## INTRODUCTION

Crush injuries to the hand often result in substantial bone and soft tissue loss, posing a significant threat to both the viability of the digits and overall hand function, particularly when the thumb is involved. As John Napier aptly described, "the hand without a thumb is at worst nothing but an animated spatula"^1^.

The treatment approach is complex. It is determined by the level of injury, extend of bone and soft tissue damage, vascularity, surgeon expertise and patient factors^2^. It requires precise decision-making which adhered to the fundamental principles and wide pool of surgical techniques. Thumb-salvage should be attempted whenever possible, using whatever techniques where expertise is available. The game plan is to maximise the hand function in the shortest period with minimum number of surgeries^3^.

Only a handful of cases reported for thumb reconstruction using this technique. Therefore, we described our case to contribute to the existing literature.

## CASE REPORT

A 60-year-old gentleman, right-hand dominant, presented with a fall from twelve-feet height while doing plumbing works at home. During the fall, his right hand hit a metal plate on the ground causing an open crush injury. Apart from the hand, no other injury was noted. He was able to ambulate and presented to the emergency department.

**Fig. 1:**
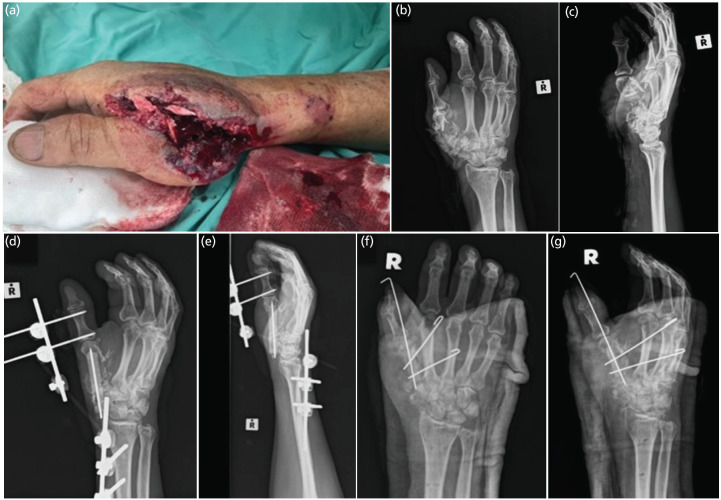
(a) Clinical photograph upon presentation. (b and c) Initial hand radiographs (anteroposterior and lateral views) showing severe comminution of the first metacarpal, trapezium and scaphoid. (d and e) Post-primary surgery images illustrating temporary filling of bone loss with antibiotic cement and stabilisation using an external fixator. (f and g) Post-definitive surgery images demonstrating implantation of an iliac bone autograft, transfixed with three k-wires.

At the casualty, he was hemodynamically stable. He suffered an open wound over the dorsum aspect of first webspace exposing torn muscles, tendons and bony fragments (Fig. 1a). The distal circulation remained brisk and sensation to the volar aspect of thumb was intact. Plain radiographs displayed substantial bone loss as per Fig. 1b and 1c.

We discussed surgical options with patient whether to salvage or primary amputation followed by delayed reconstruction. Looking into the clinical assessment and patient factors, we pursued with thumb-salvage.

The wound was debrided, antibiotic cement was inserted as a filler and external fixator applied, Fig. 1d and 1e. The wound was able to be approximated primarily. Postoperatively, the thumb remained viable. Intravenous (IV) cefuroxime was administered during the ward stay, followed by oral cefuroxime to complete a two-week course. Patient was discharged after a week of admission.

At eight weeks follow-up, the wound had healed, pin-sites were healthy and blood parameters displayed infection-free. We proceeded with the second-stage surgery utilising tricortical iliac graft and reverse-radial-forearm flap. During the approach, the induced membrane (IM) was clearly identified and incised to remove the cement filler. The IM was well-vascularised, soft, glistening, white in colour, and approximately 1mm thick, clearly demarcating the bone gap from surrounding tissue.

The bone defect measured 4cm. Both ends were denuded, the remaining first metacarpal and scaphoid. A tricortical iliac crest graft was harvested and resized. It was stabilised and transfixed to the second metacarpal (Fig. 1f and 1g). The wound then was covered using a reverse-radial-forearm flap for webspace reconstruction. The same antibiotic regimen was use as the first surgery.

Following the reconstruction, a volar-based thumb slab was applied for two weeks, followed by a well-contoured splint for three months. Rehabilitation included early protected motion at two weeks, progressing to strengthening exercises at six weeks, with an emphasis on grip strength and fine motor coordination.

**Fig. 2:**
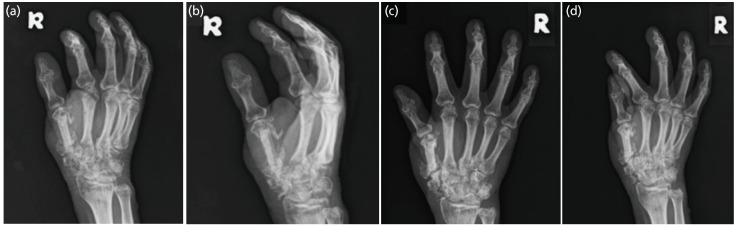
(a and b) Radiographs taken at three and (c and d) six months post-surgery showing progressive integration and union of the autograft.

**Fig. 3:**
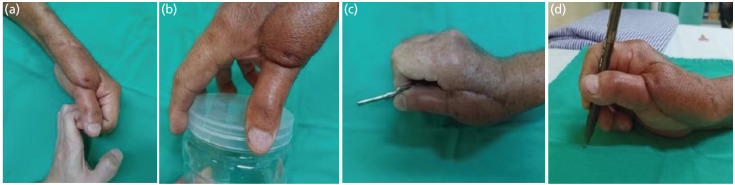
Clinical photographs at six months demonstrating preserved power grip; (a) hook grip, (b) five-jawed cradle-chuck and precision grip; (c) pad-to-side, and (d) three-jawed chuck for activities of daily living.

At four weeks follow-up, the reconstructed graft had started to fuse at both ends with preserved height and alignment. Further assessment at three and six months showed good bone incorporation, (Fig. 2). Hand functional assessment demonstrated good pad-to-side, three-jawed chuck, five-jawed cradle-chuck and hook grip (Fig. 3). Patient was happy with the outcome and able to return to work as factory operator.

## DISCUSSION

Work-related crush injuries to the hand are common in newly industrialised countries. Thumb involvement is associated with disappointing functional outcome. The principle of reconstruction is to provide a maximally functional hand in the shortest period of time with minimum amount of surgery^3^.

Initial hand assessment is crucial to determine the direction of treatment. Attending surgeon must put the utmost effort to salvage the thumb. Amputation followed by secondary reconstruction should only be considered if no function can be preserved. Reconstruction options include toe-to-thumb transfer or index pollicization with reported good functional outcome. However, hand appearance and donor-site morbidity are the drawbacks^2^. In this case, the soft tissue condition and vascularity were preserved distally, thus we strived for thumb-salvage.

Primary debridement should be thorough to avoid unnecessary repeated surgeries. During our primary surgery, critical bone defect of 4cm raised the questions of initial stabilisation and method of future reconstruction. The method of stabilisation depends on the degree of contamination. Inadequate stabilisation can lead to infection. K-wire fixation and external fixator were safe and preferred in this case^3^. If the contamination was mild, fixation with mini-plate and screws can be performed. On a case-by-case basis, primary wound closure should be performed whenever possible to protect implants and internal structures.

The secondary surgery was directed to enhance the function and appearance of the hand. Method of bone reconstruction depends on the soft tissue contamination whether it is septic or aseptic, the size of bone defect and surgeons’ expertise^2^. Autologous bone grafting whether vascularised or non-vascularised is a valid option depending on these factors. Distraction osteogenesis is well-established in the long bones. However, it was unsuitable in this case due to the absence of residual bone required for effective distraction.

In this case, the second-stage procedure was performed after eight weeks, in accordance with the recommended six-to-eight-week window by Masquelet *et al*^4^. This allowed sufficient time for maturation of the induced membrane, resolution of soft tissue inflammation, and logistical coordination. No signs of infection were present prior to the second stage.

Masquelet two-stage technique for reconstruction of metacarpals has become increasingly popular in the Western world. This technique was initially established for the lower limb and suitable in both septic and aseptic conditions. The antibiotic cement block during the first-stage acted as a gap-filler. It diffuses antibiotic locally while waiting for IM to be formed. As a mechanical pillar, it prevents fibrous tissue interposition. It forms a biological chamber which contained growth factors for maintenance and corticalization of the bone graft^2^.

Traditionally, this technique uses cancellous bone graft. However, obtaining sufficient cancellous graft to fill large defects can be challenging. While some authors have recommended adding bone substitute, a ratio greater than 1:3 has been linked to high rates of graft resorption. Incorporating an additional cortical strut, such as tricortical iliac graft, can enhance structural stability and mechanical strength, facilitating early rehabilitation and improving functional outcomes^2,4^.

Our case shares similarities with Lum *et al* in 2018, with a few important differences^2^. Both cases used the same first-stage fixation method, tricortical iliac bone grafting, and local flap coverage. The main difference was in the second-stage fixation where we used K-wires instead of a plate to help preserve interphalangeal (IP) joint movement and improve hand function.

Functional thumb is essential in both power and precision grip. Sollerman *et al* in 1995 further classified them into eight hand-grips where each pinch and grip carries certain weightage in activities of daily livings^5^. Along with hand appearance, these two goals are important to the mental wellbeing of patient. Awareness of psychological sequelae from the injury must also be integrated in the treatment follow-up.

In summary, initial hand assessment following crush injury should established the key determinants to initiate the direction of treatment, whether to salvage or amputate. The key determinants include vascularity, soft tissue contamination, size of bone defect, surgeon expertise and patient factors. Primary surgery is aimed to remove all devitalised structures and secondary surgery is aimed to improve the existing function along with appearance of the hand. The Masquelet technique is a versatile tool in addressing critical bone loss in metacarpals with satisfactory clinical outcome and hand appearance.

## CONFLICT OF INTEREST

The authors declare no potential conflict of interest.

## References

[1] Flatt AE (2002). Our thumbs.. Proc (Bayl Univ Med Cent)..

[2] Lum ZC, Park L, Huff KE, Ibrahim MA (2018). The Masquelet Technique for Thumb Metacarpal Reconstruction Following Trauma: A Case Report.. JBJS Case Connect..

[3] Lahiri A (2020). Guidelines for management of crush injuries of the hand.. J Clin Orthop Trauma..

[4] Masquelet AC, Begue T (2010). The concept of induced membrane for reconstruction of long bone defects.. Orthop Clin North Am..

[5] Sollerman C, Ejeskär A (1995). Sollerman hand function test. A standardised method and its use in tetraplegic patients.. Scand J Plast Reconstr Surg Hand Surg..

